# Gait impairment-related axonal degeneration in Parkinson’s disease by neurite orientation dispersion and density imaging

**DOI:** 10.1038/s41531-024-00654-w

**Published:** 2024-02-27

**Authors:** Xuan Wei, Shiya Wang, Mingkai Zhang, Ying Yan, Zheng Wang, Wei Wei, Houzhen Tuo, Zhenchang Wang

**Affiliations:** 1 Department of Radiology, Beijing Friendship Hospital, Capital Medical University, Beijing, China; 2 Department of Neurology, Beijing Friendship Hospital, Capital Medical University, Beijing, China; 3 Division of Science and Technology, Beijing Friendship Hospital, Capital Medical University, Beijing, China

**Keywords:** Parkinson's disease, Parkinson's disease, Diffusion tensor imaging

## Abstract

Microstructural alterations in the brain networks of Parkinson’s disease (PD) patients are correlated with gait impairments. Evaluate microstructural alterations in the white matter (WM) fiber bundle tracts using neurite orientation dispersion and density imaging (NODDI) technique in PD versus healthy controls (HC). In this study, 24 PD patients and 29 HC were recruited. NODDI and high-resolution 3D structural images were acquired for each participant. The NODDI indicators, including the intracellular neurite density index (NDI), orientation dispersion index (ODI), and isotropic volume fraction (ISO), were compared between the two groups. Diffusion-weighted (DW) images were preprocessed using MRtrix 3.0 software and the orientation distribution function to trace the main nerve fiber tracts in PD patients. Quantitative gait and clinical assessment scales were used to compare the medication “ON” and “OFF” states of PD patients. The NDI, ODI, and ISO values of the WM fiber bundles were significantly higher in PD patients compared to HC. Fiber bundles, including the anterior thalamic radiation, corticospinal tract, superior longitudinal fasciculus, forceps major, cingulum, and inferior longitudinal fasciculus, were found to be significantly affected in PD. The NDI changes of PD patients were well correlated with stride lengths in the “ON” state; ODI changes were correlated with the stride time in the “ON” and “OFF” states and ISO changes were correlated with the stride time and cadence in the “ON” state. In conclusion, combination of NODDI technique and gait parameters can help detect gait impairment in PD patients early and accurately.

## Introduction

Parkinson’s disease (PD) is the second most prevalent neurodegenerative disease^1^, characterized by bradykinesia, muscular rigidity, rest tremor, and postural and gait impairment, as well as non-motor symptoms such as cognitive deficits, depressive disorders, constipation, and sleep disorders^2^. Of these, gait impairment can severely impact the quality of life (QoL) due to the high risk of sudden falls, and inability to perform daily activities independently^3^. PD-associated typical gait impairments include sluggish movement, reduced stride length, a tendency to lean forward in the trunk, diminished postural reflexes, and lower upper limb sway^4–6^.

Gait refers to a complex sequence of body movements or postures, which allow the body to move forward in a balanced way. Gait functions are guided by the visual, cognitive, and motor functions of the organism. In healthy individuals, walking actions are physiologically controlled by the cortical and sub-cortical regions of the brain^7,8^. Since PD patients develop neuropathological symptoms related to the sub-cortical basal ganglia loops, these patients exhibit an inability to walk or move freely and a severe lack of co-ordinations between motor and cognitive actions. Many PD patients exhibited motor fluctuations. The “OFF” state was defined as the 12 h time point after taking the last dose of PD medication, while the “ON” state referred to the administration of a super-ON dose of levodopa, which corresponded to 125% of the morning levodopa equivalent dose on the same day^9^. Furthermore, although the initial gait impairment in the PD shows a positive response to the levodopa therapy, however, PD symptoms gradually become less or non-responsive due to the chronic levodopa therapy^10^. In addition, Lord et al.^11^ have identified five factors that might be associated with gait activities, namely pace, rhythm, variability, asymmetry, and postural control. Although dopaminergic medications have demonstrated their efficacies against certain aspects of gait impairment, the responses tend to be significantly variable across PD patients^12,13^.

However, the underlying pathomechanism of patient-specific gait impairment in PD has not been explored in detail^14–17^. Recent studies suggest that progressive degeneration of the white matter (WM) fiber tracts, in addition to the substantia nigra (SN) par compacta pathology, may play a significant role in the development of gait impairment in PD^18^.

Because of the non-specific nature of parameters and a lack of detailing to identify any pathologic microstructural changes, diffusion-weighted (DW) magnetic resonance imaging (MRI) techniques have been developed to troubleshoot the limitations of the conventional diffusion tensor imaging (DTI) approach for the PD brain diagnosis^19–23^. In contrast, the neurite orientation dispersion and density imaging (NODDI) technique can infer and quantify the orientation and structure of neuronal synapses by applying the directional discrete cylinder and/or the Watson distribution model, which can overcome the limitations of a regular DTI technique^24^. NODDI is a multi-compartment model that accounts for the diffusion MRI signal in each voxel as the total diffusion contributed by the diffusion restricted in axons, dendrites, extra-neurite spaces, and cerebrospinal fluid (CSF). As an advanced DW MRI technique, NODDI can also analyze microstructural changes in the WM^25^. Unlike the conventional DTI that can only provide a combined metric of multiple contributions to a voxel, NODDI can calculate specific metrics such as the neurite density index (NDI) for determining the packing density of axons or dendrites; orientation dispersion index (ODI) to assess the orientation coherence of neurites, and the isotropic volume fraction (ISO) to quantify the fraction of isotropic freely diffusing water molecules within the voxel^25^.

NODDI has been widely applied in a wide range of neuroimaging studies, including brain development^26^, neurogenetic disorders^27^, epilepsy^28^, and Alzheimer’s disease^29^. PD-related studies using NODDI have reported reduction in nigrostriatal and striatal synaptic lengths and branching in addition to loss of nigrostriatal neurons. Kamagata et al.^30^ have utilized NODDI to reveal significant reductions in NDI values of the nigrostriatal and nucleus accumbens in PD patients compared to healthy controls (HC), suggesting disease-associated microstructural changes. It has also been observed that NDI and ODI values of the nigrostriatal and nucleus accumbens are negatively correlated with the disease severity, and the NDI value of the contralateral nigrostriatal region has the highest diagnostic value for PD. Andica et al.^31^ have found that decreased contralateral distal NDI value in the nigrostriatal pathway of PD patients, indicating a decrease in the density of nerve synapses and degenerative changes in dopaminergic neurons of the nigrostriatal. Also, an elevated ISO has been observed in the posterior SN area in PD by Mitchell et al.^32^. The study found decreased NDI and increased ISO values in the frontal, temporal, limbic, and paralimbic cortical regions, corresponding to Braak stages IV–V^33^. Likewise, Guo et al. ^34^ have shown that PD patients have decreased ODI in the bilateral nigrostriatal-nigropallidal pathway as well as in the left limbic pathway in comparison to HC. Focusing on the cognitive symptoms in PD patients, Bai et al.^35^ have demonstrated that reduction in ODI values in the right frontal and bilateral caudate nuclei areas in PD patients with mild cognitive impairment compared to PD patients with normal cognition. Bange et al.^36^ have reported a positive correlation between the gait rhythm and ISO values in the right SN area. Although PD-associated changes in the brain microstructures have been well studied in NODDI, there is only one study that combines NODDI with objective gait parameters to study PD-related gait impairment.

Therefore, to comprehend the pathogenic connection between the microstructural anatomic changes in the WM and corresponding dopaminergic neuronal responses in the PD brain, we employed the NODDI technique in combination with objective gait parameters. The medication ON and OFF states of PD patients were recorded using quantitative gait and clinical scales. We hypothesize that WM microstructure alterations play an important role in inducing PD-related gait impairment.

## Results

### Demographics and behavioral analyses of the participants

The demographic and clinical data of all the participants are summarized in Table 1. PD patients and HCs were matched for age, gender, education, and Mini-Mental State Examination (MMSE) and Modified Apathy Evaluation Scale (MAES) scores. The Beck Anxiety Inventory (BAI) and Beck Depression Inventory (BDI) scores of the PD group were significantly higher than those of the HC group (Table 1). Normality tests showed that not all clinical and objective gait measurements were consistent with the normality assumption (Tables 1 and 2). Compared with the HC group, the PD group had significantly lower Berg Balance Scale (BBS) scores and higher Timed-Up and Go (TUG) values (Table 1). The velocity and step length of the HC group were higher than those of the PD group. In the PD group, the “ON” medication state significantly reduced the TUG time and scores of Movement Disorders Society-Unified Parkinson’s Disease Rating Scale, part III (MDS-UPDRS-III) (*P* < 0.01) and MDS-UPDRS-Total (*P* < 0.05) (Table 1).

**Table 1:** Demographic and clinical characteristics of the control and PD participants

Measurements	Control	PD	
		PD Clinical parameters	
		ON State	OFF State
Age	64.0 (51.5-70.0)	68.0 (63.3–70.0)	
Gender (% Male)	14 (48.3%)	10 (41.7%)	
Education ( > 9year)	15 (51.7%)	12 (50.0%)	
Duration of disease (months)	NA	52.0 (27.3–81.8)	
MMSE	29.0 (30.0–27.0)	29.0 (29.8–28.0)	
MASE	7.0 (5.0–13.0)	10.5 (4.3–17.0)	
BAI	24.0 (22.0–25.5)	26.0 (24.0–29.8)**	
BDI	3.0 (1.0–4.5)	6.0 (4.0–9.0)**	
LEDD (mg)	NA	550.0 (318.8–743.8)	
NFOGQ	NA	10.0 (41.7%)	
PDQ-39	NA	20.5 (11.5–33.3)	
MDS-UPDRS-III	NA	16.0 (12.5–23.8)	29.5 (20.0–36.8)##
MDS-UPDRS-Total	NA	39.5 (25.0–46.8)	48.0 (34.3–62.5)#
TUG	8.1 (7.3–8.6)	8.8 (8.2–10.2)**	10.5 (9.0–12.2)**#
BBS	56.0 (54.0-56.0)	52.0 (44.3–54.0)**	49.5 (39.3–52.8)**
Velocity SSP (cm/s)	117.0 (106.9–133.0)	105.2 (86.5–118.9)**	104.4 (79.1–113.5)**
Cadence SSP (step/min)	112.5 (106.6–121.4)	114.3 (105.5–119.1)	113.4 (107.7–120.5)
Stride time SSP (s)	1.1 (1.0–1.1)	1.0 (1.0–1.1)	1.1 (1.0–1.1)
Stride length SSP (cm)	127.8 (114.8–136.0)	110.9 (96.3–124.4)**	106.1 (88.7–120.4)**
Velocity FP (cm/s)	147.1 (139.6–168.4)	126.9 (109.1–142.8)**	123.7 (103.0–137.7)**
Cadence FP (step/min)	129.6 (119.7–136.4)	123.4 (114.6–132.2)	125.8 (114.9–132.3)
Stride time FP (s)	0.9 (0.9–1.0)	1.0 (0.9–1.0)	1.0 (0.9–1.0)
Stride length FP (cm)	138.3 (127.9–152.6)	122.7 (108.7–138.5)**	120.4 (101.4–129.2)**

*PD* Parkinson’s disease, *MMSE* Mini-Mental State Examination, *MASE* Modified Apathy Estimate Scale, *BAI* Back Anxiety Inventory, *BDI* Back Depression Inventory, *LEDD* levodopa equivalent daily dose (mg), *NFOGQ* New Freezing of Gait Questionnaire, *PDQ-39* Quality of life questionnaire for patients with Parkinson’s Disease, *MDS-UPDRS* International Parkinson and Movement Disorder Society-Unifified Parkinson’s Disease Rating Scale, *MDS-UPDRS-III* MDS-UPDRS motor score, *MDS-UPDRS-Total* MDS-UPDRS total score, *TUG* Timed up and Go, *BBS* Berg Balance Scale, *SSP* self-selected pace, *FP* fast pace. For control, there is no ON or OFF state. The same set of clinical data from the control was used to analyze against PD group. **P < 0.01, compared with the control group. ^#^*P* < 0.05, compared with ON State, ^##^*P* < 0.01, compared with ON State.

**Table 2:** Correlation between NODDI values and gait measures

Gait measures	NDI β/*P* value	ODI β/*P* value	ISO β/*P* value
ON			
Velocity SSP (cm/s)	0.474/0.054	−0.405/0.099	0.092/0.663
Cadence SSP (step/min)	0.267/0.285	−0.265/0.294	0.247/0.267
Stride time SSP (s)	−0.301/0.216	0.351/0.155	−0.294/0.177
Stride length SSP (cm)	0.485/**0.049**	−0.423/0.086	−0.002/0.991
OFF			
Velocity SSP (cm/s)	0.447/0.074	−0.334/0.177	0.083/0.699
Cadence SSP (step/min)	0.298/0.225	−0.304/0.222	0.274/0.212
Stride time SSP (s)	−0.323/0.173	0.405/0.095	−0.334/0.117
Stride length SSP (cm)	0.434/0.085	−0.307/0.219	−0.039/0.857
ON			
Velocity FP (cm/s)	0.367/0.129	−0.367/0.132	0.287/0.180
Cadence FP (step/min)	0.301/0.177	−0.390/0.088	0.471/**0.024**
Stride time FP (s)	−0.301/0.165	0.444/**0.049**	−0.495/**0.015**
Stride length FP (cm)	0.350/0.161	−0.340/0.178	0.154/0.482
OFF			
Velocity FP (cm/s)	0.405/0.104	−0.245/0.321	0.174/0.421
Cadence FP (step/min)	0.370/0.112	−0.437/0.067	0.348/0.096
Stride time FP (s)	−0.363/0.110	0.483/**0.040**	−0.384/0.062
Stride length FP (cm)	0.350/0.171	−0.150/0.555	0.058/0.795

*β* Standardized regression coefficients, *ON* in medication ON state, *OFF* in medication OFF state, *SSP* self-selected pace, *FP* fast pace. *NDI* neurite density index, *ODI* orientation dispersion index, *ISO* isotropic volume fraction. The bold values highlight the significant correlation coefficients between objective gait parameters and the NODDI values.

### Tract-Based Spatial Statistics (TBSS) analysis

**Fig. 1:**
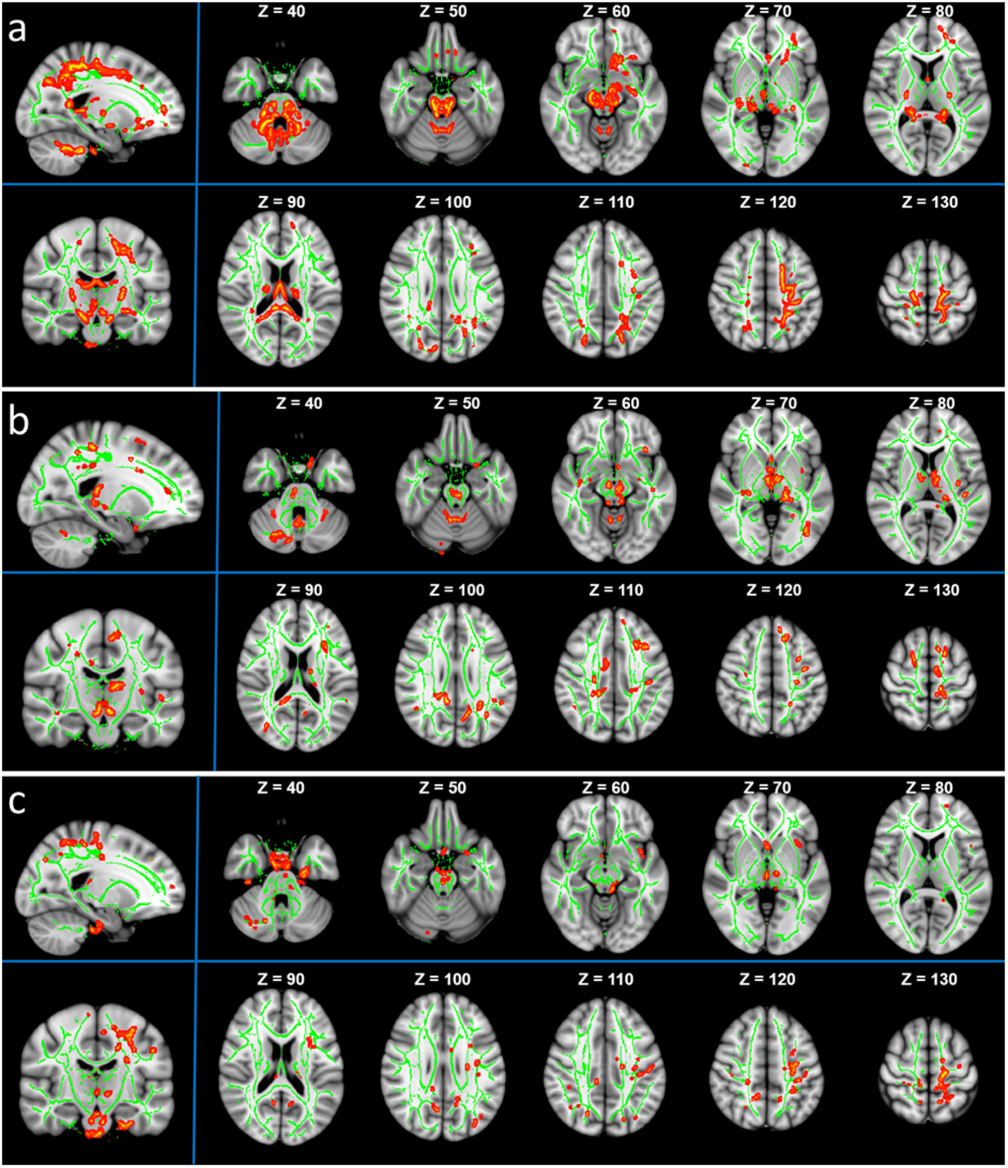
Altered white matter microstructure in PD by Tract-Based Spatial Statistics (TBSS) analysis. **a** Voxel-wise TBSS analysis results of neurite density index (NDI) images between the PD and the healthy control (HC) groups. Significant voxel-wise group differences are shown in red for metrics that are increased in patients (*P* < 0.05, corrected for multiple comparisons with Threshold-Free Cluster Enhancement). Results are overlaid on axial sections of the group-specific white matter (WM) skeleton (shown in green). The left side of the image corresponds to the right hemisphere of the brain. The WM areas with significant differences in NDI values include anterior thalamic radiation, corticospinal tract, forceps major, left inferior fronto-occipital fasciculus, and left superior longitudinal fasciculus. **b** Voxel-wise TBSS analysis of orientation dispersion index (ODI) images between the PD and the HC groups. Significant voxel-wise group differences are shown in red for metrics that are increased in patients (*P* < 0.05, corrected for multiple comparisons with Threshold-Free Cluster Enhancement). Results are overlaid on axial sections of the group-specific WM skeleton (shown in green). The left side of the image corresponds to the right hemisphere of the brain. The WM areas with significant differences in ODI values include anterior thalamic radiation, right cingulum (cingulate gyrus), corticospinal tract, forceps major, and left superior longitudinal fasciculus. **c** Voxel-wise TBSS analysis results of isotropic volume fraction (ISO) images between the PD and the HC groups. Significant voxel-wise group differences are shown in red for metrics that are increased in PD patients (*P* < 0.05, corrected for multiple comparisons with Threshold-Free Cluster Enhancement). Results are overlaid on axial sections of the group-specific WM skeleton (shown in green). The left side of the image corresponds to the right hemisphere of the brain. The WM areas with significant differences in Iso values include the left corticospinal tract, left superior longitudinal fasciculus, left Cingulum (hippocampus), and left anterior thalamic radiation.

Between the PD and the control groups, there were significant differences in broad fibers (*P* < 0.05 corrected by Threshold-Free Cluster Enhancement; Fig. 1). The WM areas with significantly different NDI values included anterior thalamic radiation, corticospinal tract, forceps major, left inferior fronto-occipital fasciculus, and left superior longitudinal fasciculus (Fig. 1a). Likewise, WM areas with significantly different ODI values involved the anterior thalamic radiation, right cingulum (cingulate gyrus), corticospinal tract, forceps major, and left superior longitudinal fasciculus (Fig. 1b). And WM areas with significantly varying ISO values included the left corticospinal tract, left superior longitudinal fasciculus, left cingulum (hippocampus), and left anterior thalamic radiation (Fig. 1c).

**Fig. 2:**
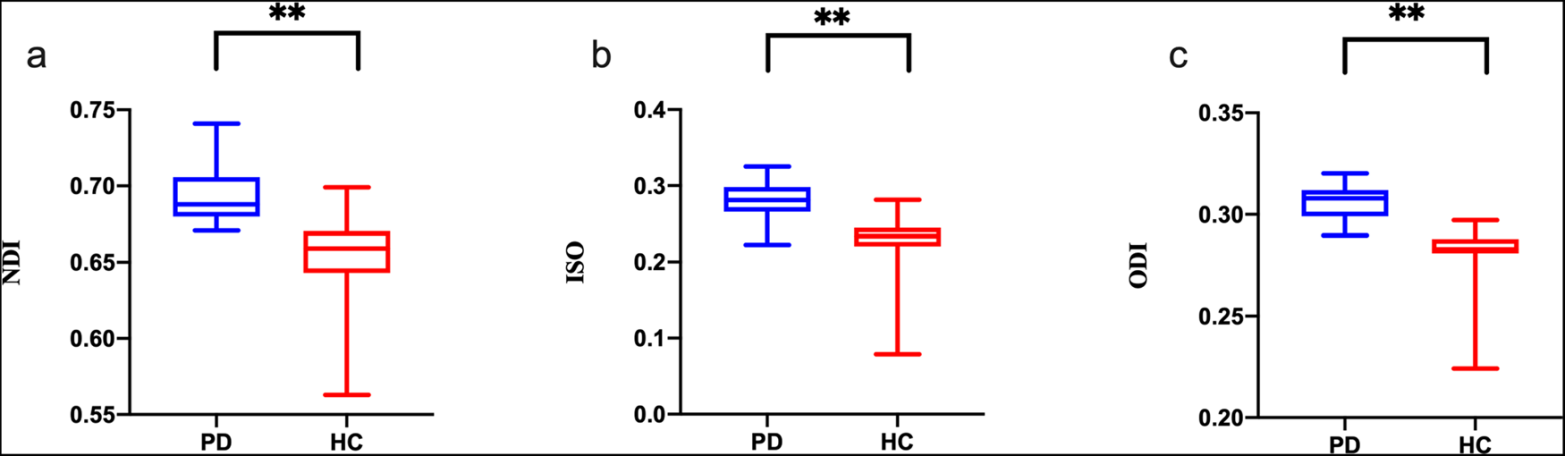
Compared with the PD group, the NODDI metrics (NDI, ODI, and ISO) were significantly decreased across multiple WM areas in the HC group. **a** Compared with the HC group, the PD group demonstrated a significant increase in NDI values. **b** Compared with the HC group, the PD group demonstrated a significant increase in ISO values. **c** Compared with the HC group, the PD group demonstrated a significant increase in ODI values. **P < 0.01. All elements of boxplots: (i) centre line: median; (ii) upper bound of box: 75th percentile; (iii) bottom bound of box: 25th percentile; (iv) upper whisker: maximum value; (v) lower whisker: minimum value. NDI neurite density index, ODI orientation dispersion index, ISO isotropic volume fraction.

As shown in Fig. 2, the NDI, ODI, and ISO values of their specific bundle of fibers were significantly increased in PD patients compared to their HC counterparts, and differences remained significant even after adjusting for age and gender (*P* < 0.05).

### Correlation analysis

**Fig. 3:**
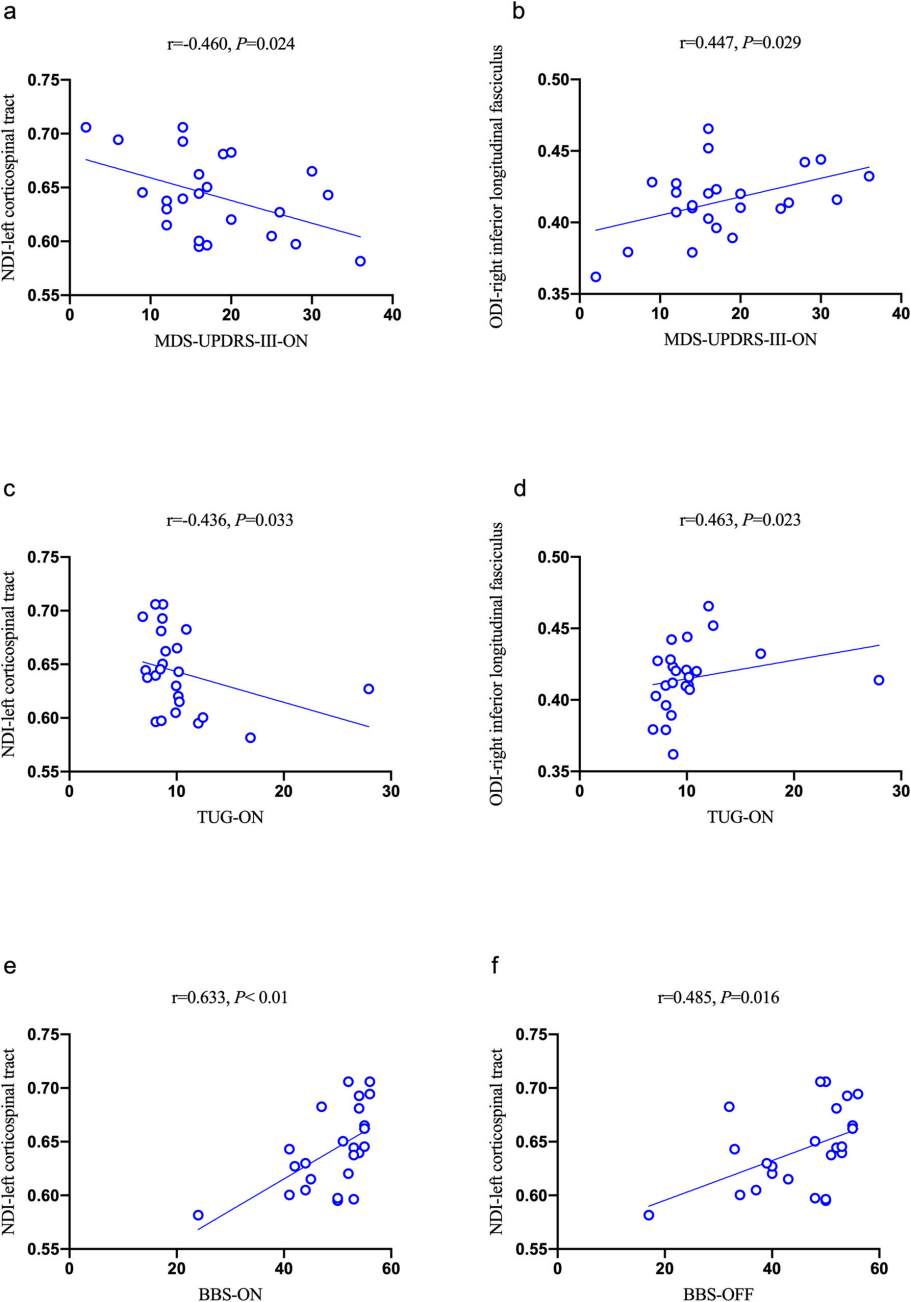
Associations between NODDI metrics and clinical scales in PD. **a** The NDI value of the left corticospinal tract was negatively correlated with MDS-UPDRS-III-ON score (r = −0.460, *P* = 0.024). **b** The ODI value of right inferior longitudinal fasciculus was positively correlated with MDS-UPDRS-III-ON score (r = 0.447, *P* = 0.029). **c** The NDI value of the left corticospinal tract was negatively correlated with TUG-ON score (r = −0.436, *P* = 0.033). **d** The ODI value of right inferior longitudinal fasciculus was positively correlated with TUG-ON score (r = 0.463, *P* = 0.023). **e** The NDI value of left corticospinal tract was positively correlated with BBS-ON score (r = 0.633, *P* < 0.01). **f** The NDI value of left corticospinal tract was positively correlated with BBS-OFF score (r = 0.485, *P* = 0.016). MDS-UPDRS-III-ON, the Part III of International Parkinson and Movement Disorder Society-Unified Parkinson’s Disease Rating Scale during the “ON” state; TUG-ON, Timed-Up and Go during the “ON” state; BBS-ON, Berg Balance Scale during “ON” state; BBS-OFF, Berg Balance Scale during “OFF” state.

The NDI value of left corticospinal tract was negatively correlated with the MDS-UPDRS-III-ON and TUG-ON scores (*r* = -0.460, *P* = 0.024 & *r* = -0.436, *P* = 0.033; Fig. 3) but was positively correlated with either BBS-ON or BBS-OFF score (*r* = 0.633, *P* < 0.01 or *r* = 0.485, *P* = 0.016; Fig. 3). The ODI value of the right inferior longitudinal fasciculus was positively correlated with both MDS-UPDRS-III-ON and TUG-ON scores (*r* = 0.447, *P* = 0.029 & *r* = 0.463, *P* = 0.023; Fig. 3). There were no significant correlations between the NODDI model metric and BDI, BAI, MMSE, MAES, Parkinson’s Disease Questionnaire-39 (PDQ-39), MDS-UPDRS-III-OFF, MDS-UPDRS-Total, TUG-OFF, or New Freezing of Gait Questionnaire (NFOGQ).

Table 2 displays the standardized regression coefficients for several NODDI measures that were correlated strongly with the objective gait parameters in PD patients. In the “ON” state, a higher NDI value during self-selected pace (SSP) was significantly associated with a greater stride length (β = 0.485, *P* = 0.049; Table 2). Furthermore, at fast pace (FP), a higher ODI value was significantly correlated with a longer stride time in both the “ON” and “OFF” states in PD patients (β = 0.444, *P* = 0.049 & β = 0.483, *P* = 0.040; Table 2). While in the “ON” state, ISO values were positively correlated with the cadence (β = 0.471, *P* = 0.024; Table 2) and negatively correlated with the stride time (β = −0.495, *P* = 0.015; Table 2) at FP.

## Discussion

This study utilized the NODDI approach to investigate the microstructural changes in the WM of PD brains. It is the first NODDI imaging report to use TBSS for WM microstructure changes in PD patients with gait impairment. The major findings of this study were (1) consistent with previous findings, the alteration of NDI, ODI, and ISO occurred in broad WM microstructure in PD subjects compared to HCs; (2) some significant areas of the WM were correlated with scores of MDS-UPDRS-III, TUG, BBS during the “ON” and “OFF” states, and specific objective gait parameters.

NODDI, a new multi-compartment diffusion-weighted imaging (DWI) data analysis technique, can be used to assess the complexity of the axonal and dendritic microstructures based on the following parameters: (1) NDI for synaptic density; (2) ODI for determining directional changes in nerve prominent, and (3) ISO (aka extracellular free water) for quantifying the proportion of CSF in each voxel^25,37^. Like previous studies, we observed an increased ISO values of PD patients compared to HC, which might indicate an axonal degeneration phenotype, characterized by persistent water accumulation and increased membrane spacing due to neuronal loss^30^. However, unlike most other studies, we found increased NDI and ODI values in PD patients. The reduced NDI may reflect axonal loss or disorganization^25^. However, Ogawa et al.^38^ suggest that a significant increase in NDI values may be affected by iron accumulation and/or gliosis in the damaged site. Moreover, an overdose of levodopa therapy may elevate NDI and ODI values in gait-impaired PD patients^39^. On the other hand, we postulate that the underlying mechanism could be a compensatory one in PD patients. Alternatively, such conflicting findings may be due in part to differences in sample characteristics (e.g., disease duration or staging) and enrollment protocols. Therefore, additional histopathological studies are necessary to elucidate the underlying mechanisms responsible for increasing NDI and ODI values. Furthermore, the accumulation of α-synuclein in PD has been shown to occur in axons and is closely related to the degradation of proteins related to or essential for the axonal transport^40^. Our study found NODDI indicators of WM fiber bundles showed significant changes, supporting axonal pathology may be the central factor for WM microstructural changes in PD^41^.

Gait impairment is a significant clinical problem in PD but its underlying mechanisms are not fully understood. Our study revealed microstructural changes in six fiber bundles—anterior thalamic radiation, corticospinal tract, superior longitudinal fasciculus, forceps major, cingulum, and inferior longitudinal fasciculus, which could be associated with gait impairment in PD, consistent with previous reports. Thus, this study may help elucidate the neuropathological basis of gait impairment in PD. Vercruysse et al.^42^ have identified that PD-associated microstructural changes may propagate through the subcortical WM layers, including the intra-hemispheric cortico-cortical association fibers of the superior longitudinal fasciculus, the motor-related corticofugal tract and thalamic radiation. Verlinden et al.^43^ reported microstructural changes in the thalamic radiations, association tracts, and forceps major strongly associated with gait impairment in PD. Significant structural changes in the inferior longitudinal fasciculus (right hemisphere) in PD patients with freezing of gait were observed by Pietracupa et al.^44^. Given that gait impairment is a complex sequence of biological actions involving the motor, cognitive, and visual systems, the cingulum might be involved in the regulation of rapid body movement, affecting the gait^45^.

PD patients were assessed for their motor functions using clinical scales and gait measures to correlate with imaging outcomes. We found that as the NDI value of the left corticospinal tract decreased, the PD patient exhibited lower BBS scores and higher MDS-UPDRS-III-ON and TUG-ON scores. And in the case of the right inferior longitudinal fasciculus, MDS-UPDRS-III-ON and TUG-ON scores increased proportionally with that of ODI, suggesting a correlation between nerve axon integrity and the severity of motor symptoms in PD. Also, we observed a correlation between microstructural alterations in the WM and the clinical scale scores in PD patients in the “ON” state, suggesting that this correlation may exist independently of dopamine levels^46^. These results suggest that altered NDI values of the left corticospinal tract and ODI values of the right inferior longitudinal fasciculus may be useful for evaluating gait impairment in synucleinopathies.

Regarding gait parameters, PD patients had significantly lower velocity and stride length in comparison to the HC. When comparing gait parameters between the “ON” and “OFF” states of PD patients, a non-significant difference was observed, possibly due to the fact that gait is affected by not only dopaminergic signals but also other signals such as cholinergic signals^47^. Importantly, correlations were found between gait measures (velocity, cadence, stride time, and stride length) and NODDI indicators, such that a higher ODI value in the right cingulum correlated with a higher stride time, suggesting that microstructural disruption in this region may underly gait impairment in PD^41^. Change in the NDI value in the anterior thalamic radiation was correlated with the stride length in the “ON” state of PD patients at SSP, and the ISO change in the left corticospinal tract was correlated with both the cadence and stride time in the “ON” state at FP. Considering the compensatory mechanism of PD pathology, these correlations might be related to compensatory changes in the NODDI indicators in the later stage of the disease, while limited compensation does not reflect any visible gait improvements^46^. In summary, all these correlations between various microstructural parameters and gait improvement in the “ON” state in PD patients suggest that levodopa may partially activate these designated brain regions, thus manifesting a positive response to the levodopa therapy^46^. Additionally, we observed correlations between gait parameters at SSP versus FP. Specifically, the right cingulum’s ODI was positively correlated with the FP stride time but not with the SSP stride time. Therefore, FP gait parameters may indicate gait performance reserve, and any pathological changes in these parameters may predict motor disability^48^.

No significant correlations were found between age, gender, education, MMSE score, or MAES score in the PD and HC groups. However, PD patients had significantly higher BAI and BDI scores than the HC group. Depression and anxiety symptoms, which are related to WM alteration, occur frequently in PD patients. Radiation fibers emanating from the thalamus may be critical to the pathogenesis of depressive disorders in PD patients^49^. However, we could not find any significant correlations between the NODDI measures and the BAI or BDI score.

Previous NODDI studies investigated pathological signs in the SN and WM fiber bundles, but few explored the correlation between the NODDI measures and detailed objective gait parameters in PD patients. There is only one study conducted by Bange et al.^36^ that combines the ISO and ODI of NODDI with treadmill-measured gait parameters to investigate the motor characteristics of PD patients. Notably, the major merit of this study is that it’s a comprehensive clinical and objective gait assessment using computerized measurements in both medications “ON” and “OFF” states. Additionally, TBSS analysis was introduced to further strengthen the observations.

Also, this study suffers from certain limitations. First, the number of participants in each group was relatively smaller. In future studies, we will enroll a statistically large number of subjects and also expand the involvement of PD study centers. Second, we could not complete the NODDI sequence in the medication “OFF” state, mainly due to the physically challenging conditions of the elderly subjects.

Although our study has some limitations, our findings are valuable and offer new insights into PD research. Given the interest in establishing a more reliable imaging portfolio of gait impairment at earlier stages of PD, it is important to determine whether a combination of NODDI metrics and gait parameters can detect microstructural changes in PD brains at the pre-symptomatic stage. In future studies, we plan to combine other structural and functional MRI techniques to enhance our understanding of PD-associated brain microstructural changes and establish a more reliable imaging portfolio of gait impairment in neurodegenerative diseases. Furthermore, whether exercise interventions can improve WM microstructural integrity remains an open-ended question. Future studies would be benefited from this imaging portfolio to investigate whether exercise can modulate gait performance in PD.

In summary, these correlations between the NODDI metrics and gait parameters suggest that synaptic dysfunction and subsequent neurodegeneration may synergistically contribute to the pathophysiology of gait impairment in PD. Our findings suggest that microstructural abnormalities in specific fiber bundles of the WM may be closely related to gait and have the potential to serve as imaging markers for the early and precise identification of gait impairment in PD patients, thereby providing meaningful imaging information for early identification intervention. This may ultimately provide imaging markers for the early and precise identification of gait impairment in PD patients, thereby providing meaningful imaging information for early intervention.

## Methods

### Participants

A total of 24 patients who were diagnosed with PD were recruited from the Movement Disorders Program at the Beijing Friendship Hospital Capital Medical University. These patients were treated with standard doses of dopaminergic medications. To be included in this study, patients had to be clinically diagnosed with idiopathic PD based on the UK Parkinson’s Disease Society Brain Bank criteria^50^. While patients were excluded if they had atypical or secondary Parkinsonism; confounding neurological or psychiatric disorder(s); any conditions that might prevent their abilities to give informed consent, and other neurological diseases leading to movement disorders. Also, patients with dementia and/or having metallic implants were considered unsafe for MRI examinations. Besides, we recruited 29 gender- and age-matched HC from the community. All participants provided written informed consent. The demographics and clinical characteristics of all the recruited individuals are described in Table 1.

### Ethics statement

This study was approved by the ethics committees of Beijing Friendship Hospital, Capital Medical University(2019-P2-283-02) and conducted by the Declaration of Helsinki. And all subjects have signed informed consent forms.

### Clinical assessments

PD patients with gait impairments were identified from their medical history and using MDS-UPDRS part-II, and part-III (a score ≥1 on item 2.12 or 2.13 in MDS-UPDRS-II or a score ≥1 on item 3.10 or 3.11 in MDS-UPDRS-III). Other assessments of motor functions in the “ON” and “OFF” medication states were full MDS-UPDRS, BBS, NFOGQ, and the TUG. For all participants, objective gait parameters were assessed using a 20-foot-long computerized Zeno Walkway Gait Analysis System (Proto Kinetics, Havertown, PA, USA) at a SSP and a FP. The QoL was assessed using PDQ-39. Cognitive performances were assessed using the MMSE^51^. Anxiety and depression disorders were evaluated using the BDI score, BAI score, and MAES (Table 1).

### MRI data acquisition and preprocessing

DW images were acquired on a 3.0 T MRI system (Prisma, Siemens, Erlangen, Germany) with a 64-channel phase-array head coil. The major acquisition parameters were set as the field-of-view (FOV) = 209 × 209 mm^2^, matrix size (MS) = 116 × 116 mm^2^, slices = 84 (with no gap), voxel size (VS) = 1.8 × 1.8 × 1.8 mm^3^, repetition time (TR) = 3000 ms, echo time (TE) = 81 ms and flip angle = 90°.

For each subject, two-shell high angular resolution diffusion imaging (HARDI) data were acquired, including 1 non-DW image (b = 0 s/mm^2^, B0) with phase-encoding in the anterior-posterior (AP) direction, 10 non-DW images (b = 0 s/mm^2^) with phase-encoding in the posterior-anterior (PA) direction, 64 DW images from 64 non-collinear gradient directions with a b-value of 1000 s/mm^2^ and a phase-encoding in the PA direction, and 64 DW images from 64 non-collinear gradient directions with a b-value of 2000 s/mm^2^ and phase-encoding in the PA direction.

The 3D T1-weighted image for each subject was obtained using a Magnetization Prepared Rapid Gradient-Echo (MPRAGE) sequence with the following imaging parameters, FOV = 224 × 256 mm^2^, MS = 448 × 512 mm^2^, 192 sagittal slices, VS = 0.5 × 0.5 × 1 mm^3^, TR = 2530 ms, TE = 2.98 ms, and a flip angle = 7°.

### MRI data preprocessing and NODDI processing

The DW images were preprocessed using an optimized processing pipeline introduced by Maximov, et al.^52^. First, noise and Gibbs-ringing corrections were performed for the DWI data using the Marchenko–Pastur principle component analysis (MP-PCA)^53^ and the method of local, subvoxel-shifts^54,55^. Then non-DW images with opposite phase encoding directions were used to correct the echo-planar imaging (EPI) geometric distortion with the function of *topup*^56,57^ offered in FSL version 6.0.3 (https://fsl.fmrib.ox.ac.uk/fsl/fslwiki/topup)^58^. The diffusion MRI data of each individual were visually inspected by two specialists to make sure there were no apparent artifacts arising from acquisition or data processing procedures. Additionally, Distortions that appeared due to eddy-current, head motion, and susceptibility-originated artifacts were corrected by topup and eddy^59^ together in FSL v6.0.3. Subjects with translation > 3 mm or rotation > 3° in any direction were excluded. B1 bias field inhomogeneity correction for DWI data was performed using *dwibiascorrect* in MRtrix 3.0^55^. Finally, 11 preprocessed non-DW images were averaged to generate the mean B0 image for the downstream processing. Consequently, the preprocessed DWI data included 1 B0 image, 64 DWI images with a b-value of 1000 s/mm^2^, and 64 DWI images with a b-value of 2000 s/mm^2^.

3D T1-weighted images were segmented using the CAT12 toolbox (Computational Anatomy Toolbox for SPM) (http://www.neuro.uni-jena.de/cat/index.html) to generate the brain image in T1 space (brain_T1). DTI metrics, including the fractional anisotropy (FA) map, were calculated with the *dtifit* function in FSL^58^. NODDI metrics were estimated using the NODDI toolbox (http://www.nitrc.org/projects/noddi_toolbox), including NDI, ODI, and (Gaussian) ISO parameters.

### Statistical analysis

Demographic and clinical characteristics of PD and HC individuals were described as the percentage (%) of categorical variables and median of interquartile ranges (IQRs) of continuous variables. Categorical variables were analyzed by chi-square (χ^2^) test and continuous variables by Student’s *t*-test or Wilcoxon rank-sum test. The disease-relevant NODDI measures and clinical scales (MDS-UPDRS-III scores, TUG scores, and BBS scores) were assessed by calculating Pearson’s or Spearman’s rank correlation, as appropriate. Tests of normality showed that not all clinical and objective gait measurement data were eligible for the assumption of normality.

The Wilcoxon rank-sum test was used to compare gait parameters between PD patients and HCs. For PD patients, changes in objective gait parameters between the “ON” and “OFF” states were analyzed using the paired two-sample Wilcoxon rank-sum test. Correlations between the NODDI indicators and gait parameters were analyzed using multiple linear regression (MLR) models, adjusted for age and sex, in the PD and HC groups, respectively. The standardized regression coefficient (β) and the corresponding *P* value were calculated. Statistical analyses were performed using SPSS version 25.0 software (SPSS, Inc., Chicago, IL).

TBSS^60^ from FSL was used to investigate the whole brain WM abnormality patterns in PD patients. First, FA images were analyzed to estimate the mean FA skeleton following the TBSS steps. Then NDI, ODI, and ISO values were projected onto the original mean FA skeleton to generate a 4D projected dataset using the *tbss_non_FA* in FSL. Finally, voxelwise statistics on the skeletonized NODDI metrics between the HC and the PD groups were carried out using the *randomize* tool, which is a permutation-based inference tool for nonparametric statistics implemented in FSL, including age and gender as covariates (50000 permutations, corrected for multiple comparisons with Threshold-Free Cluster Enhancement^61^, *P* < 0.05).

### Supplementary information

Reporting-summary

## Data Availability

The corresponding authors offer accessibility to the data utilized in this study, ensuring their availability upon the submission of reasonable inquiries. It is important to note that, to safeguard privacy, precautions may be implemented to regulate access to sensitive data.

## Code availability

NA
