# Supplementary material for: Gait impairment-related axonal degeneration in Parkinson’s disease by neurite orientation dispersion and density imaging
Source: NPJ Parkinsons Dis. 2024 Feb 27;10:45. doi: 10.1038/s41531-024-00654-w (PMC10899173; doi:10.1038/s41531-024-00654-w)
Supplement: Supplementary file 1 — Reporting-summary [file 41531_2024_654_MOESM1_ESM.pdf]

nature portfolio

Corresponding author(s): Zhenchang Wang; Houzhen Tuo

Last updated by author(s): Jan 12, 2024

Reporting Summary

Nature Portfolio wishes to improve the reproducibility of the work that we publish. This form provides structure for consistency and transparency in reporting. For further information on Nature Portfolio policies, see our Editorial Policies and the Editorial Policy Checklist.

Statistics

For all statistical analyses, confirm that the following items are present in the figure legend, table legend, main text, or Methods section.

n/a	Confirmed	
☐	☒	The exact sample size (*n*) for each experimental group/condition, given as a discrete number and unit of measurement
☐	☒	A statement on whether measurements were taken from distinct samples or whether the same sample was measured repeatedly
☐	☒	The statistical test(s) used AND whether they are one- or two-sided<br>*Only common tests should be described solely by name; describe more complex techniques in the Methods section.*
☐	☒	A description of all covariates tested
☐	☒	A description of any assumptions or corrections, such as tests of normality and adjustment for multiple comparisons
☐	☒	A full description of the statistical parameters including central tendency (e.g. means) or other basic estimates (e.g. regression coefficient) AND variation (e.g. standard deviation) or associated estimates of uncertainty (e.g. confidence intervals)
☐	☒	For null hypothesis testing, the test statistic (e.g. F, t, r) with confidence intervals, effect sizes, degrees of freedom and P value noted<br>*Give P values as exact values whenever suitable.*
☒	☐	For Bayesian analysis, information on the choice of priors and Markov chain Monte Carlo settings
☒	☐	For hierarchical and complex designs, identification of the appropriate level for tests and full reporting of outcomes
☒	☐	Estimates of effect sizes (e.g. Cohen's *d*, Pearson's *r*), indicating how they were calculated

Our web collection on statistics for biologists contains articles on many of the points above.

Software and code

Policy information about availability of computer code

Data collection	No software was used
Data analysis	SPSS version 25.0 software (SPSS, Inc., Chicago, IL), FSL version 6.0.3 (https://fsl.fmrib.ox.ac.uk/fsl/fslwiki/topup), CAT12 toolbox (Computational Anatomy Toolbox for SPM) (http://www.neuro.uni-jena.de/cat/index.html), NODDI toolbox (http://www.nitrc.org/projects/noddi_toolbox), MRtrix 3.0.

For manuscripts utilizing custom algorithms or software that are central to the research but not yet described in published literature, software must be made available to editors and reviewers. We strongly encourage code deposition in a community repository (e.g. GitHub). See the Nature Portfolio guidelines for submitting code & software for further information.

Data

Policy information about availability of data

All manuscripts must include a data availability statement. This statement should provide the following information, where applicable:

- Accession codes, unique identifiers, or web links for publicly available datasets
- A description of any restrictions on data availability
- For clinical datasets or third party data, please ensure that the statement adheres to our policy

As the data in our study includes identifiable human research participants and sensitive information, the data supporting the findings of this study are available from the corresponding author upon reasonable request. Reasonable requests: 1. Academic collaboration: Other researchers or academic institutions may request

access to the raw data for collaborative research or to validate the study findings. 2. Academic review: During the peer review process, reviewers may request access to the raw data to assess the credibility and accuracy of the research. 3. Audit requirements: Certain organizations or government agencies may require access to the raw data for auditing or compliance purposes. Instances where raw data will not be provided: 1. Privacy protection: The raw data may contain personally identifiable information or sensitive data, and to protect the privacy of participants, the raw data will not be provided. 2. Legal restrictions: There may be legal regulations or contractual agreements that limit the sharing of raw data. 3. Data ownership: The raw data may be protected by intellectual property rights or considered as proprietary information, and the raw data will not be provided to avoid infringement of rights.

Research involving human participants, their data, or biological material

Policy information about studies with human participants or human data. See also policy information about sex, gender (identity/presentation), and sexual orientation and race, ethnicity and racism.

Reporting on sex and gender	Our study conclusions are not limited to a specific sex or gender. Furthermore, sex and gender were not considered in the study design. Gender data were recorded based on biological characteristics and self-reports of the participants. All participants had no abnormalities in terms of gender identity and biological sex.
Reporting on race, ethnicity, or other socially relevant groupings	All participants included in this study were of Asian descent and born in China. The ethnic background of all participants was Han Chinese, and there were no other sociological characteristics present that could impact the analysis of results.
Population characteristics	The demographic and clinical data of all the participants are summarized in Table 1. With regard to the diagnostic grouping, we categorized all participants into a Parkinson's disease(PD) group and a healthy control(HC) group based on their diagnoses. PD patients and HCs were matched for age, gender and education.
Recruitment	A total of 24 patients who were diagnosed with PD were recruited from the Movement Disorders Program at the Beijing Friendship Hospital Capital Medical University.Besides, we recruited 29 genderand age-matched HC from the community.
Ethics oversight	The ethics committees of Beijing Friendship Hospital, Capital Medical University

Note that full information on the approval of the study protocol must also be provided in the manuscript.

Field-specific reporting

Please select the one below that is the best fit for your research. If you are not sure, read the appropriate sections before making your selection.

☒ Life sciences ☐ Behavioural & social sciences ☐ Ecological, evolutionary & environmental sciences

For a reference copy of the document with all sections, see nature.com/documents/nr-reporting-summary-flat.pdf

Life sciences study design

All studies must disclose on these points even when the disclosure is negative.

Sample size	Our participants consisted of two groups, patients with Parkinson's disease and healthy controls, totaling 53 participants. They were selected from 135 candidates based on the quality and completeness of the data, and the degree of matching of the relevant covariates. They were the remaining samples after the screening, so no sample size calculation was performed.
Data exclusions	Excluded subjects without quantitative gait assessment and without DWI and sMRI and subjects who with poor image quality.
Replication	This study is an observational study that primarily aims to investigate the association between gait characteristics and imaging features. Therefore, experimental reproducibility is not applicable in this study.
Randomization	This study did not employ randomization as it is an observational study that divided participants into a patient group and a healthy control group based on disease diagnosis. Covariates such as gender, age, and educational level were matched between the two groups. Therefore, randomization is not applicable in this study.
Blinding	Our study does not involve blinding as it is an observational study without any related interventions.

Reporting for specific materials, systems and methods

We require information from authors about some types of materials, experimental systems and methods used in many studies. Here, indicate whether each material, system or method listed is relevant to your study. If you are not sure if a list item applies to your research, read the appropriate section before selecting a response.

Materials & experimental systems

n/a	Involved in the study
☒	☐ Antibodies
☒	☐ Eukaryotic cell lines
☒	☐ Palaeontology and archaeology
☒	☐ Animals and other organisms
☒	☐ Clinical data
☒	☐ Dual use research of concern
☒	☐ Plants

Methods

n/a	Involved in the study
☒	☐ ChIP-seq
☒	☐ Flow cytometry
☐	☒ MRI-based neuroimaging

Plants

Seed stocks

Report on the source of all seed stocks or other plant material used. If applicable, state the seed stock centre and catalogue number. If plant specimens were collected from the field, describe the collection location, date and sampling procedures.

Novel plant genotypes

Describe the methods by which all novel plant genotypes were produced. This includes those generated by transgenic approaches, gene editing, chemical/radiation-based mutagenesis and hybridization. For transgenic lines, describe the transformation method, the number of independent lines analyzed and the generation upon which experiments were performed. For gene-edited lines, describe the editor used, the endogenous sequence targeted for editing, the targeting guide RNA sequence (if applicable) and how the editor was applied.

Authentication

Describe any authentication procedures for each seed stock used or novel genotype generated. Describe any experiments used to assess the effect of a mutation and, where applicable, how potential secondary effects (e.g. second site T-DNA insertions, mosiacism, off-target gene editing) were examined.

Magnetic resonance imaging

Experimental design

Design type: Resting state

Design specifications: This study is not applicable.

Behavioral performance measures: This study is not applicable.

Acquisition

Imaging type(s): Structural MRI and diffusion MRI

Field strength: 3.0 T

Sequence & imaging parameters: DW images were acquired on a 3.0T MRI system (Prisma, Siemens, Erlangen, Germany) with a 64-channel phase-array head coil. The major acquisition parameters were set as the field-of-view (FOV) = 209 × 209mm2, matrix size (MS) = 116 × 116mm2, slices = 84 (with no gap), voxel size (VS) = 1.8 × 1.8 × 1.8mm3, repetition time (TR) = 3000ms, echo time (TE) = 81ms and flip angle = 90°. The 3D T1-weighted image for each subject was obtained using a Magnetization Prepared Rapid Gradient-Echo (MPRAGE) sequence with the following imaging parameters, FOV = 224 × 256mm2, MS = 448 × 512mm2, 192 sagittal slices, VS = 0.5 × 0.5 × 1mm3, TR = 2530ms, TE = 2.98ms, and a flip angle = 7°.

Area of acquisition: Whole brain

Diffusion MRI ☒ Used ☐ Not used

Parameters: Two-shell high angular resolution diffusion imaging (HARDI) data were acquired, including 1 non-DW image (b = 0s/mm2, B0) with phase-encoding in the anterior-posterior (AP) direction, 10 non-DW images (b = 0s/mm2) with phase-encoding in the posterior-anterior (PA) direction, 64 DW images from 64 non-collinear gradient directions with a b-value of 1000s/mm2 and a phase-encoding in the PA direction, and 64 DW images from 64 non-collinear gradient directions with a b-value of 2000s/mm2 and phase-encoding in the PA direction.

Preprocessing

Preprocessing software: FSL v6.0.3, MRtrix 3.0

Normalization: Fractional anisotropy images were normalized to the FMRIB58_FA standard-space image with non-linear registration method.

Normalization template: MNI152

Noise and artifact removal	First, noise and Gibbs-ringing corrections were performed for the DWI data using the Marchenko–Pastur principle component analysis (MP-PCA and the method of local, subvoxel-shifts. Then non-DW images with opposite phase encoding directions were used to correct the echo-planar imaging (EPI) geometric distortion with the function of topu offered in FSL version 6.0.3. Distortions that appeared due to eddy-current, head motion, and susceptibility-originated artifacts were corrected by topup and eddy together in FSL v6.0.3.
Volume censoring	FSL; Subjects with translation > 3 mm or rotation > 3° in any direction were excluded.

Statistical modeling & inference

Model type and settings	General linear model
Effect(s) tested	Two-Sample Unpaired T-Test

Specify type of analysis: ☒ Whole brain ☐ ROI-based ☐ Both

Statistic type for inference (See Eklund et al. 2016)	Voxel-wise
Correction	Threshold-Free Cluster Enhancement

Models & analysis

n/a	Involved in the study
☒	☐ Functional and/or effective connectivity
☒	☐ Graph analysis
☒	☐ Multivariate modeling or predictive analysis
